# Structural, electrical and hydrogen sensing properties evaluation of fabricated PdO thin films by thermal oxidation

**DOI:** 10.1038/s41598-026-49588-8

**Published:** 2026-05-06

**Authors:** Murat Ozabaci, Esme Isik, Necmettin Kilinc

**Affiliations:** 1 Scientific and Technological Research Center, Inonu University, Malatya, 44280 Türkiye; 2 Department of Electronics and Automation, Malatya Turgut Ozal University, Malatya, 44700 Türkiye; 3 Institute of Solid State Physics, University of Latvia, Kengaraga Street 8, Riga, LV-1063 Latvia; 4 Department of Physics, Faculty of Science & Arts, Inonu University, Malatya, 44280 Türkiye

**Keywords:** PdO thin films, Sensors, Hydrogen detection, Thermal oxidation, Thickness effect, Pd, Reduction, Chemistry, Materials science, Nanoscience and technology, Physics

## Abstract

Palladium oxide (PdO) thin films (~ 60 nm thick) were grown on quartz substrates using a two-step process involving DC magnetron sputtering of Pd films followed by thermal oxidation in dry air at 450–750 °C for 4 h. In this work, a systematic temperature-dependent oxidation strategy is employed to directly correlate phase evolution, microstructural transformation, thickness effects, and hydrogen sensing properties of PdO thin films, an aspect that has not been extensively addressed in the existing literature. Structural, morphological, and optical characterizations confirmed full Pd-to-PdO conversion above 550 °C, with crystallite sizes increasing up to 30 nm. AFM and FESEM showed porous, domain-like morphologies, and UV-Vis analysis indicated direct band gaps in the range of 2.06–2.42 eV. XPS verified the presence of stoichiometric PdO with Pd²⁺ oxidation states. Electrical measurements revealed semiconducting behavior with thermally activated conduction, yielding activation energies between 0.045 and 0.092 eV depending on annealing temperature. The sensor response showed no significant dependence on the operating temperature, and the film annealed at 750 °C exhibited the highest sensing response at 400 ppm H₂. The Pt-contacted device detected 1000 ppm H₂ at room temperature, demonstrating stable operation without elevated temperatures. Room-temperature hydrogen sensing tests showed that PdO exhibits two distinct sensing mechanisms: a reversible p-type surface reaction at low H₂ concentrations (≤ 1000 ppm) and a reduction-driven response at high concentrations (2%), where PdO is chemically transformed into metallic Pd, leading to a sharp resistance drop and metallic-like conduction. Beyond temperature variation, PdO thin films with thicknesses of 5, 10, and 20 nm were also fabricated and annealed at 650 °C to examine the influence of film thickness on hydrogen sensing performance.

## Introduction

Hydrogen sensors are becoming increasingly important in technology due to the widespread use of hydrogen gas in various fields such as the chemical industry, metallurgy, electronics, biomedical, aerospace, and food processing. Hydrogen also presents a significant potential to be used as a clean energy source, especially in the future of the transportation industry^1–3^. However, due to the unusual properties of hydrogen compared to other combustible or flammable gases such as butane, propane, methane, and acetone, there are some technical challenges and risks in its operation and storage as well as production and transportation^4,5^. The fact that hydrogen has the smallest density and atomic radius among all kinds of gases makes it highly diffusive and enhances the probability of leakage when used, for example, as an energy source^6^. Moreover, since it is colorless and odorless, it cannot be detected by human senses. In addition to its high diffusion coefficient (0.61 cm^2^/s in the air), it is extremely flammable and explosive in a wide range of concentrations (4%–75%, v/v) with its low ignition temperature (520–580 °C) and low minimum ignition energy (0.017 mJ)^7–10^. These make it necessary to develop a reliable, stable and highly sensitive hydrogen sensing system to quantify hydrogen concentration in real-time and prevent possible accidents, explosions, and hazardous effects on human health.

In recent years, hydrogen sensors with platforms based mainly on optical, resistive, acoustic, electrochemical, catalytic, thermal conductivity, mechanical, magnetic, and work function-based sensing mechanisms have been the subject of numerous research efforts^11–14^. Most of these platforms utilize Palladium (Pd) due to its exceptional ability to reversibly absorb hydrogen up to 900 times its volume^15^. Upon exposure, the dissociation and diffusion of hydrogen atoms into the Pd lattice trigger a transformation into palladium hydride (PdH_x_). This process causes a significant lattice expansion (up to 6%) and a corresponding increase in electrical resistivity (up to 80%), which serves as the fundamental detection mechanism for resistive sensors^16–19^. These structural changes occur both on the surface and within interstitial sites such as grain boundaries and voids^20,21^.

Despite the promising property of Pd in capturing hydrogen atoms, there are a number of performance limitations that need to be overcome to achieve a more efficient hydrogen sensor. For this purpose, the use of palladium alloys such as Pd-Au, Pd-Ag, Pd-Ni, or palladium oxide (PdO) instead of pure Pd is among the modifications frequently applied in recent years^22–25^. As compared to the Pd sensors, PdO-based sensors are less prone to irreversible surface contamination, especially due to the deposition of sulfuric compounds (e.g., H_2_S and SO_2_) and CO, as they operate at higher temperatures^26^. Also, PdO mitigates the damage known as hydrogen embrittlement, which occurs as a result of repeated exposure of the sensor to hydrogen over multiple cycles^27,28^. This phenomenon causes the mechanical stability of the sensor to deteriorate through the formation of permanent nano/micro-cracks and blisters between grains. On the other hand, PdO-based sensors indicate lower sensitivity to hydrogen due to the less reactive nature of the oxide layers that act as barriers against hydrogen absorption and diffusion^29^. Nevertheless, depending on application needs and working conditions, the higher anti-poisoning performance and better mechanical stability that PdO-based sensors have make it an attractive candidate for more stable and durable hydrogen sensors^30–32^.

The motivation for this study is further supported by the recent 2024 review by Badica and Lorinczi^27^, which highlights that although the conversion of palladium (Pd) into palladium oxide (PdO) is well established, systematic and comparative evaluations of PdO thin film properties remain relatively limited. While several studies have reported PdO-based sensing and catalytic applications, comprehensive investigations that directly correlate controlled oxidation parameters with structural evolution and functional hydrogen sensing performance are still limited. Moreover, as emphasized in the literature, obtaining well-defined and stable PdO phases and H_2_ sensors through thermal oxidation is a delicate process, since stoichiometry, crystallinity, phase stability, and H_2_ sensing response are highly sensitive to processing temperature and thickness of the structures^33–39^. These temperature and thickness sensitivities limit a comprehensive understanding of the interplay between oxidation conditions, microstructural development, and the resulting hydrogen sensing behavior.

In this study, 60 nm thick Pd thin films were deposited on quartz substrates via DC magnetron sputtering and subsequently annealed in dry air at four different temperatures (450 °C, 550 °C, 650 °C, and 750 °C) for 4 h to induce controlled oxidation into PdO. The selection of the 60 nm film thickness and the 450–750 °C annealing range was based on structural stability and phase transformation considerations reported in previous studies^33–35,39,40^. A thickness of 60 nm was chosen to ensure continuous and morphologically stable film formation on the quartz substrate, thereby minimizing island-like growth and thermally induced agglomeration frequently observed in ultra-thin metallic films^41^. In addition, to examine the effect of film thickness on hydrogen sensing performance, additional PdO thin films with thicknesses of 5, 10, and 20 nm were fabricated and annealed at 650 °C. Regarding thermal treatment, 450 °C was considered the threshold for effective phase transformation from metallic Pd to crystalline PdO. The upper limit of 750 °C was selected in accordance with the reported thermal stability window of PdO, as the oxide phase begins to decompose toward metallic Pd at temperatures approaching 800–850 °C^27,42,43^. Therefore, this temperature interval enables a systematic investigation of PdO formation, microstructural evolution, and phase stability within its most reliable thermal regime.

The primary objective of this work was to evaluate the suitability of the applied growth and oxidation parameters for producing high-quality PdO thin films and to assess their performance as resistive hydrogen sensors. To achieve this, the temperature-dependent evolution of structural, morphological, optical, elemental, chemical-state, electrical, and hydrogen sensing properties was comprehensively examined using X-ray diffraction (XRD), field emission scanning electron microscopy coupled with EDX (FE-SEM/EDX), atomic force microscopy (AFM), UV–Vis spectrophotometry, X-ray photoelectron spectroscopy (XPS), and resistive hydrogen sensing measurements.

## Experimental methods

The fabrication of PdO thin films was carried out in two stages in the present work. Firstly, Pd films with a thickness of 60 nm were deposited on quartz substrates using the DC sputtering technique. A high-purity palladium target (99.99%, 0.25 mm thick and 2 inches in diameter) mounted on a circular magnetron was used in the sputtering system (NANOVAK, NVBJ300 PVD System). After the base pressure of the chamber reached 2 × 10^− 6^ Torr, about 5 sccm high-purity argon (99.999%) was introduced to the chamber in order to obtain a 5 mTorr deposition pressure. The substrates were placed at a distance of 90 mm from the Pd target and were rotated at 10 rpm during the deposition process at a DC power of 25 W. The deposition rate was kept constant at 0.05 nm/s throughout the deposition process. The thickness of prepared thin films was monitored by an Inficon VGC501 with a quartz crystal microbalance (QCM). The as-deposited Pd films were then exposed to high-purity dry air (99.999%) in a tubular furnace at a flow rate of 200 sccm to convert Pd to PdO. The thermal annealing was realized at four different temperatures, 450 °C, 550 °C, 650 °C, and 750 °C for 4 h at a heating and cooling rate of 5 °C/min. Hereafter, the films with different annealing temperatures will be denoted as PdO-450, PdO-550, PdO-650, and PdO-750.

**Fig. 1:**
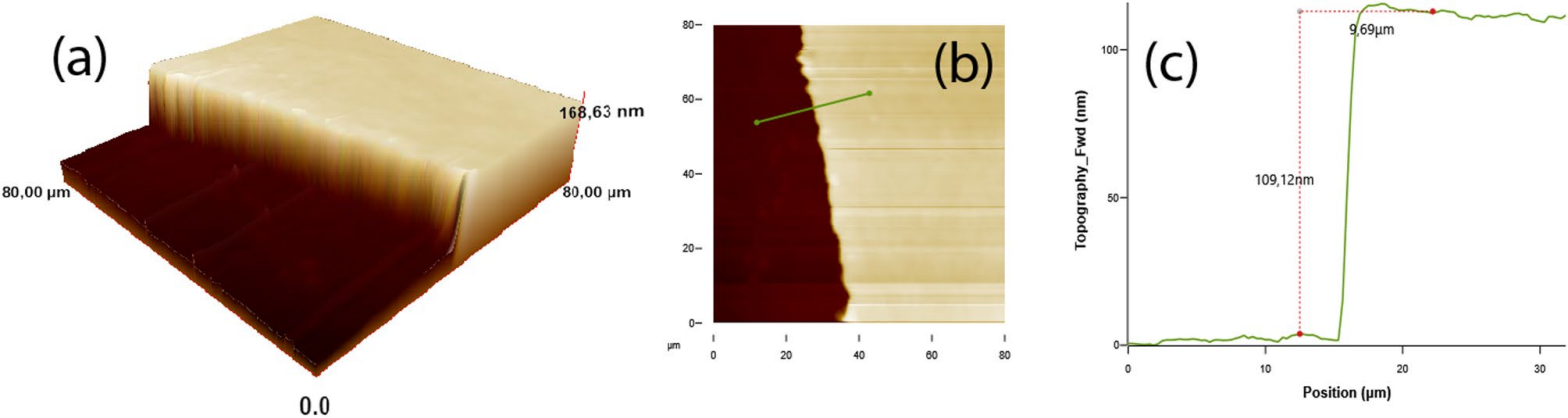
(**a**) AFM 3D image, (**b**) AFM topographic image and (**c**) corresponding line profile of the thickness of the 100 nm thick Pd film fabricated based on Inficon QCM.

Before the deposition of 60 nm thick Pd films, a 100 nm thick Pd film (based on the Inficon QCM) was fabricated in the above-mentioned sputtering system to check the accuracy of the Inficon QCM. The thickness of the 100 nm thick Pd film was measured with a Nanomagnetics Instruments scanning hall probe microscope operating in atomic force microscopy mode (SHPM-AFM). The obtained topographic and 3D images showed that the deviation of the Inficon QCM in determining the coating thickness was less than 10% as indicated in Fig. 1a-c. Additionally, Nanomagnetics Instruments’ SHPM-AFM was also used to investigate the topography and roughness of PdO films.

X-ray diffraction patterns of the fabricated PdO films were acquired with a Rigaku D/Max-2200 X-ray diffractometer utilizing Cu-Kα1 (40 kV, 40 mA, λ = 1.5405 Å) radiation with 2θ between 5° and 80° at a scan rate of 3°/min. The structural morphologies were examined by both a Zeiss-Gemini SEM 300 field emission scanning electron microscope (FE-SEM) and LEO-Evo 40 scanning electron microscope (SEM) attached with a Bruker 125 eV energy-dispersive X-ray spectroscope (EDX). A Jasco V-770 double-beam UV-Visible (UV-Vis) spectrophotometer was employed to study the optical properties of the PdO films.

The DC electrical measurements were performed with Keysight B2901BL Precision Source Measure Unit in a homemade aluminum cylindrical cell that has a gas inlet-outlet, a heatable sample holder with a Pt100 temperature sensor, and electrical feedthroughs. The temperature of the samples was varied from room temperature to 160 °C by using a Lakeshore 335 temperature controller. Before placing the PdO films into the measurement cell, two rectangular-shaped (5 × 2 mm^2^) silver contacts were thermally evaporated on the PdO films by leaving a 5 mm gap to serve as electrodes. To determine the DC electrical properties, current-voltage (I-V) measurements were made under dry airflow in the range of −1 V to 1 V, depending on temperature. The resistance measurements of PdO films monitored continuously by the Keithley 2700 Multimeter, and the Alicat mass flow controllers delivered the desired amount of hydrogen and dry air as a carrier gas to the chamber for hydrogen sensing tests. In order to evaluate the effect of thickness on the hydrogen sensing performance of PdO thin films, 5, 10, and 20 nm Pd films were coated on the quartz substrates using with the same method, and then these films were annealed at 650 °C for 4 h to obtain the PdO film. The hydrogen concentration was varied from 20 ppm to 2%, and the operation temperature of the sensors was changed from room temperature to 150 °C. Some of the characterization results of the 60 nm PdO thin films are summarized in Table 1.

**Table 1:** Summary of oxidation parameters and the corresponding microstructural, optical, and electrical properties of 60 nm Pd/PdO thin films deposited on quartz substrates by DC magnetron sputtering.

Sample name	Oxidation paramaters	Crystallite size (nm)	RMS roughness (nm)	Band gap (eV)	Activation energy (meV)	Dislocation density x10^15^ m^− 2^	Micro strain x10^− 3^
Pd	As-deposited	16.7	–	–	–	3.59	6.14
PdO-450	450 °C, 4 h, dry air	14.6	0.73	2.06	–	4.69	8.17
PdO-550	550 °C, 4 h, dry air	23.2	2.22	2.17	44.8	1.86	5.3
PdO-650	650 °C, 4 h, dry air	23.9	10.2	2.42	92.1	1.75	5.14
PdO-750	750 °C, 4 h, dry air	30.4	3.21	2.16	52.7	1.08	4.14

## Results and discussion

**Fig. 2:**
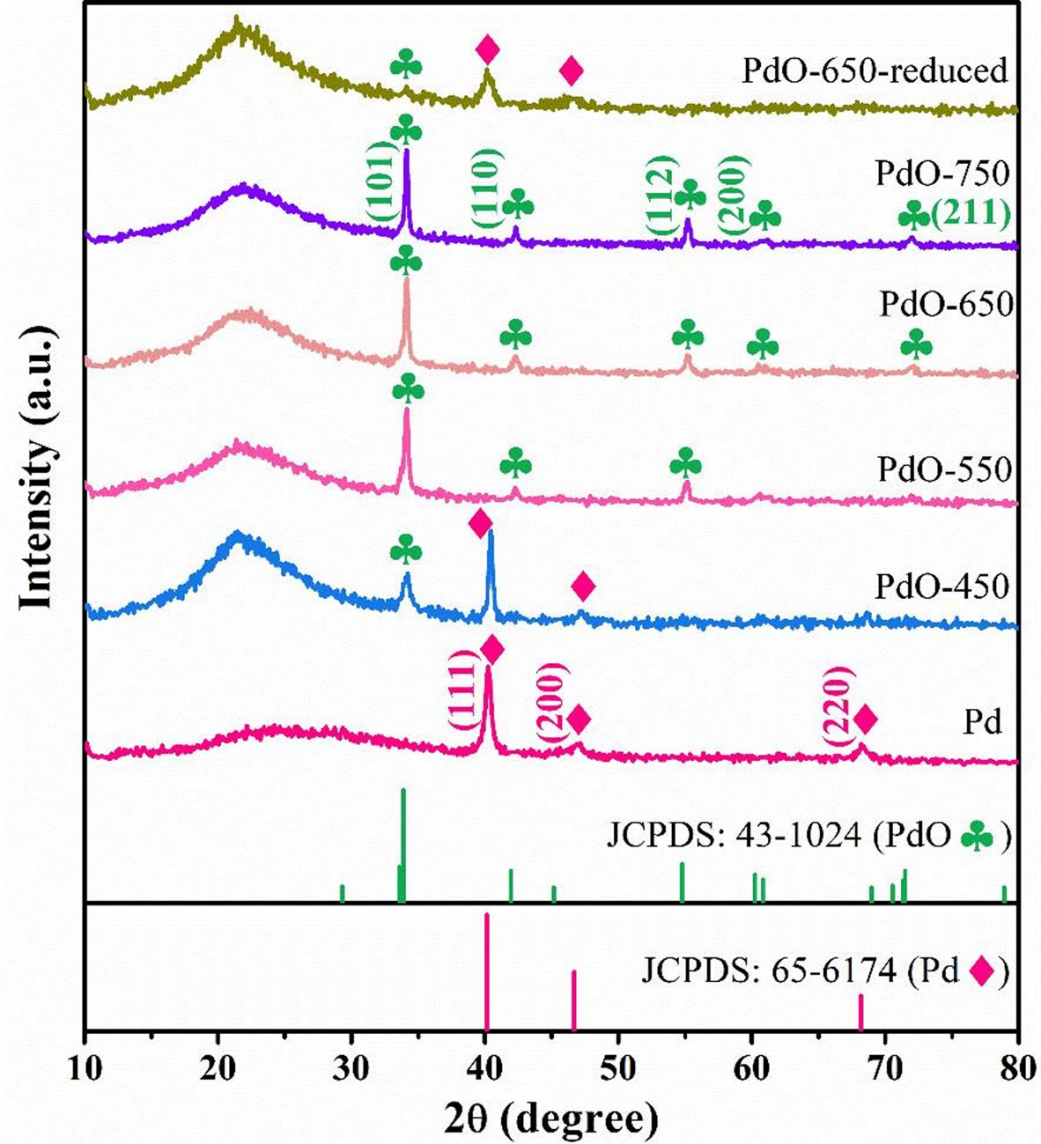
XRD patterns of Pd, PdO-450, PdO-550, PdO-650, PdO-750, and PdO-650 after hydrogen reduction.

The X-ray diffraction patterns of Pd and PdO films are shown in Fig. 2 along with the corresponding phases and (*hkl*) Miller indices. The XRD patterns show that PdO formation partially begins when the Pd film is subjected to thermal annealing at 450 °C. However, the presence of Pd peaks in the pattern of PdO-450 indicates that thermal annealing of Pd films at 450 °C is not sufficient for the complete conversion of Pd to PdO. Upon the increase of the thermal annealing temperature to 550 °C, it is easily seen that Pd-related peaks in the pattern completely vanish and PdO peaks appear. A further increase of the annealing temperature to 650 °C and subsequently to 750 °C did not lead to any significant change in the pattern except the formation of more noticeable minor peaks of PdO, which is an indication of the presence of more crystalline PdO nanoparticles in the films^44^. After reduction under 2% hydrogen for more than two hours at room temperature, the PdO-650 film was completely converted to a Pd film, as clearly evidenced by the XRD spectrum in the green line. The significant disappearance of the PdO peaks and the distinct emergence of the Pd peaks in the PdO-650-reduced sample strongly demonstrate that this process is highly reversible. After the reduction, the XRD pattern exhibits a noticeable decrease in peak intensity accompanied by significant peak broadening (increased FWHM). This behavior suggests a reduction in crystallite size and/or an increase in microstrain and structural disorder, indicating partial deterioration of the crystalline structure. It is proposed that H₂ molecules dissociate into atomic hydrogen on the PdO surface, forming surface hydroxyl (O–H) groups; subsequently, water (H₂O) is generated and may remain strongly bound to oxygen vacancies or other reduction sites^45^.

The crystallite sizes of the films were estimated by means of the Scherrer formula (1) by fitting the FWHM and 2θ values of the dominant peak belonging to the (101) crystal plane and were found to be 14.6, 23.2, 23.9, and 30.4 nm for the PdO-450, PdO-550, PdO-650, and PdO-750 films, respectively (Table 1). A similar analysis was performed for the Pd and PdO-650-reduced samples using the (111) reflection, yielding crystallite sizes of 16.7 and 10.4 nm, respectively, corroborating the previous interpretation. Dislocation density (δ) and microstrain (ε) values were also calculated and tabulated in Table 1 by using Eqs. (2) and (3), where D is the crystallite size, *λ* is the X-ray wavelength, β is the full width at half maximum (FWHM), and θ is the diffraction angle of the peak^46^. As expected, both parameters decreased inversely with increasing crystallite size. This trend can be associated with the enhanced atomic mobility at elevated oxidation temperatures, which facilitates domain growth and recrystallization processes. Consequently, atoms acquire sufficient energy to diffuse and reorganize into larger crystallites, leading to a reduction in lattice defects and internal strain. The 2θ and relative intensity of the peaks obtained from the PdO films closely match the standard data file (JCPDS Card No. 43–1024), which has tetragonal symmetry with lattice parameters a = 3.043, b = 3.043, and c = 5.337 Å and the P42/mmc space group.

1 $$\:D=\frac{0.94\lambda\:}{\beta\:Cos\theta\:}$$

2 $$\:\delta\:=\frac{1}{{D}^{2}}\:$$

3 $$\:\epsilon\:=\frac{\beta\:}{4tan\theta\:}$$

In addition to temperature, the oxygen partial pressure (pO_2_) plays a decisive role not only in the oxidation rate but also in determining which oxide phase is thermodynamically stabilized during the transformation. As reported in previous surface phase diagram studies of Pd under an O₂ atmosphere, low oxygen partial pressures favor the formation of surface oxide structures, whereas higher pO₂ conditions promote the stabilization and growth of bulk PdO. This indicates that oxygen chemical potential governs the phase selection during oxidation. Therefore, under reduced oxygen partial pressure, the system may remain in a surface-oxide regime or exhibit Pd/PdO phase coexistence, while sufficiently high pO₂ facilitates complete bulk PdO formation^47,48^. In the present study, oxidation was performed in flowing dry air, which provides a relatively high and stable oxygen chemical potential, thus favoring full Pd-to-PdO conversion above 550 °C.

**Fig. 3:**
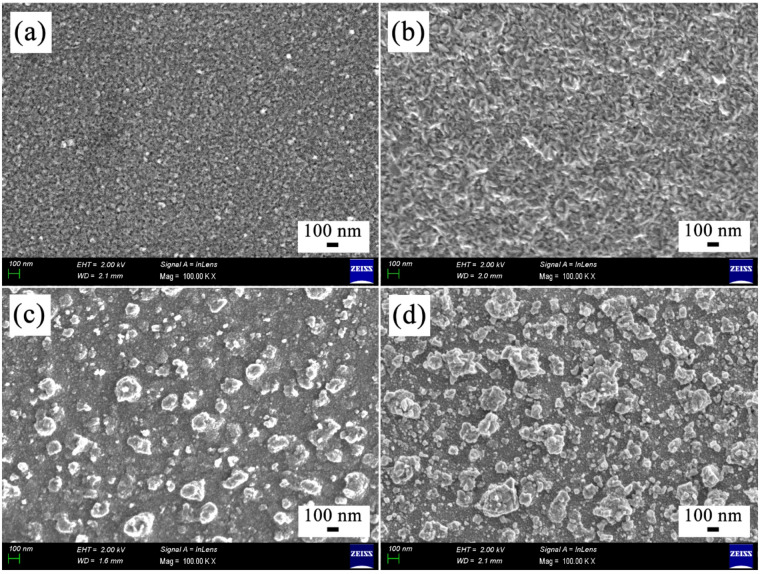
FESEM images of (**a**) PdO-450, (**b**) PdO-550, (**c**) PdO-650, and (**d**) PdO-750 films.

FESEM images of PdO films are illustrated in Fig. 3a-d. It can be easily noticed from the figure that the surface morphologies of the films change significantly depending on the thermal annealing temperature. One can see from Fig. 3a that the PdO-450 film has a smooth surface, and the domain structure cannot be clearly resolved within the resolution limits of the instrument. It can be said that the thermal annealing of the Pd film at 450 °C leads to a partial conversion from Pd to PdO nanoparticles when evaluated together with the XRD results. When oxidation was carried out at 550 °C, it was observed that the surface structure evolved to a slightly porous structure with minor peaks and valleys. This structure can be attributed to the coalescence and increased density of PdO nanoparticles. Furthermore, since the porous structure enlarges the surface area of the film exposed to oxygen, such morphology can promote the nucleation and growth of PdO domains and can be considered as a previous stage of PdO domain formation. Thus, increasing the oxidation temperature to 650 °C led to a dramatic change in the surface morphology by eliminating the low-amplitude layered-like structure and introducing the powder-like circular domains and randomly arranged PdO agglomerates with non-uniform geometry. This morphology implies the presence of thicker and rougher oxide layers in the structure. From Fig. 3c, the diameter of the domains is estimated to be in the range of 1–10 nm, while the size of the non-uniform agglomerates reaches around 200 nm. FESEM images of PdO-750 show a similar morphology to that of PdO-650 in terms of the presence of powder-like circular domains and irregularly shaped agglomerated particles. However, the concentration and size of the agglomerates are slightly higher in PdO-750 than in PdO-650. Additionally, the relatively more definite geometrical shape of the agglomerates observed on PdO-750 suggests that the crystallization is appreciably better on PdO-750 compared to PdO-650, which is in agreement with the XRD results. Considering the general structure of the oxidized films, it can be said that all films have a compact and uniform structure. EDX analyses of the films confirmed the formation of PdO on the surfaces of all PdO films but did not reveal any significant difference regarding the Pd/O ratio.

**Fig. 4:**
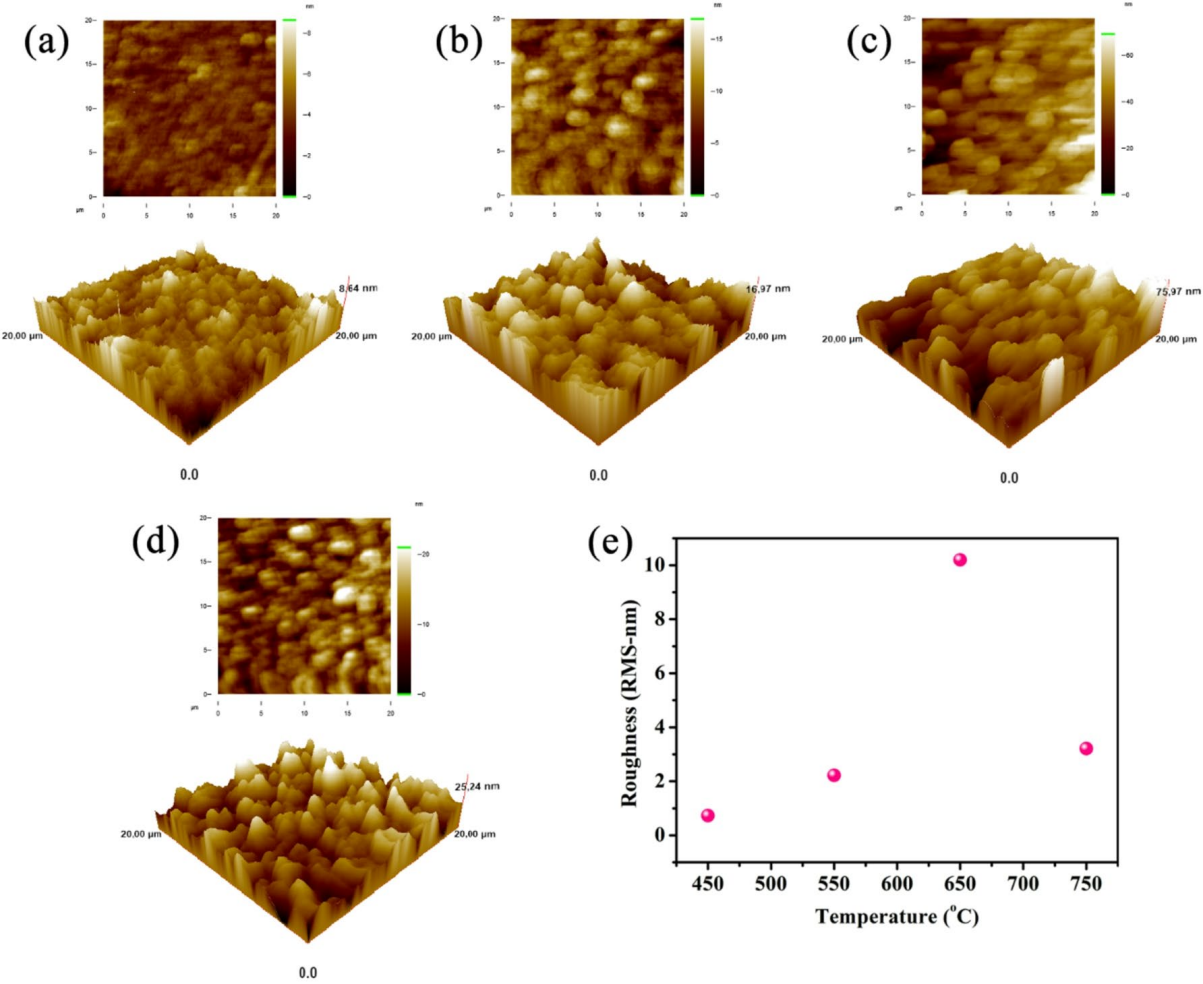
AFM topographic images and AFM 3D images of (**a**) PdO-450, (**b**) PdO-550, (**c**) PdO-650, and (**d**) PdO-750 films. (**e**) The RMS roughness of the films oxidized at 450 °C, 550 °C, 650 °C and 750 °C.

Figure 4a–d shows the topographic and 3D AFM images of four different oxidized films. These images exhibit the surface morphology of the films as well as their surface roughness. The root mean square (RMS) roughness of the films was obtained as 0.73 nm, 2.22 nm, 10.2 nm, and 3.21 nm for PdO-450, PdO-550, PdO-650, and PdO-750 films, respectively, as plotted in Fig. 4e. The significant increase in roughness with increasing oxidizing temperature up to 650 °C confirms the conversion of Pd atoms to oxide grains, clusters, or agglomerates. The observed peak in surface roughness at 650 °C followed by a decrease at 750 °C can be explained by a grain coalescence-driven microstructural evolution model. During oxidation up to 650 °C, rapid Pd-to-PdO conversion induces volumetric expansion and stress development within the film, promoting oxide nucleation and the formation of porous, non-coalesced PdO clusters. This leads to a highly roughened surface morphology. However, at 750 °C, enhanced atomic diffusion and grain boundary mobility facilitate grain coalescence and the filling of inter-grain voids, resulting in microstructural densification and partial surface smoothing driven by surface energy minimization. This transformation can be noticed in the 3D images in Fig. 4c-d, reflecting the replacement of the coarse-grained structure in PdO-650 with the finer-grained structure in PdO-750. These results agree well with the above-mentioned FESEM and XRD analyses. The roughness values reveal that thermal annealing of Pd films at 650 °C produces much higher porosity and, hence, a larger surface area within the applied four different temperatures.

**Fig. 5:**
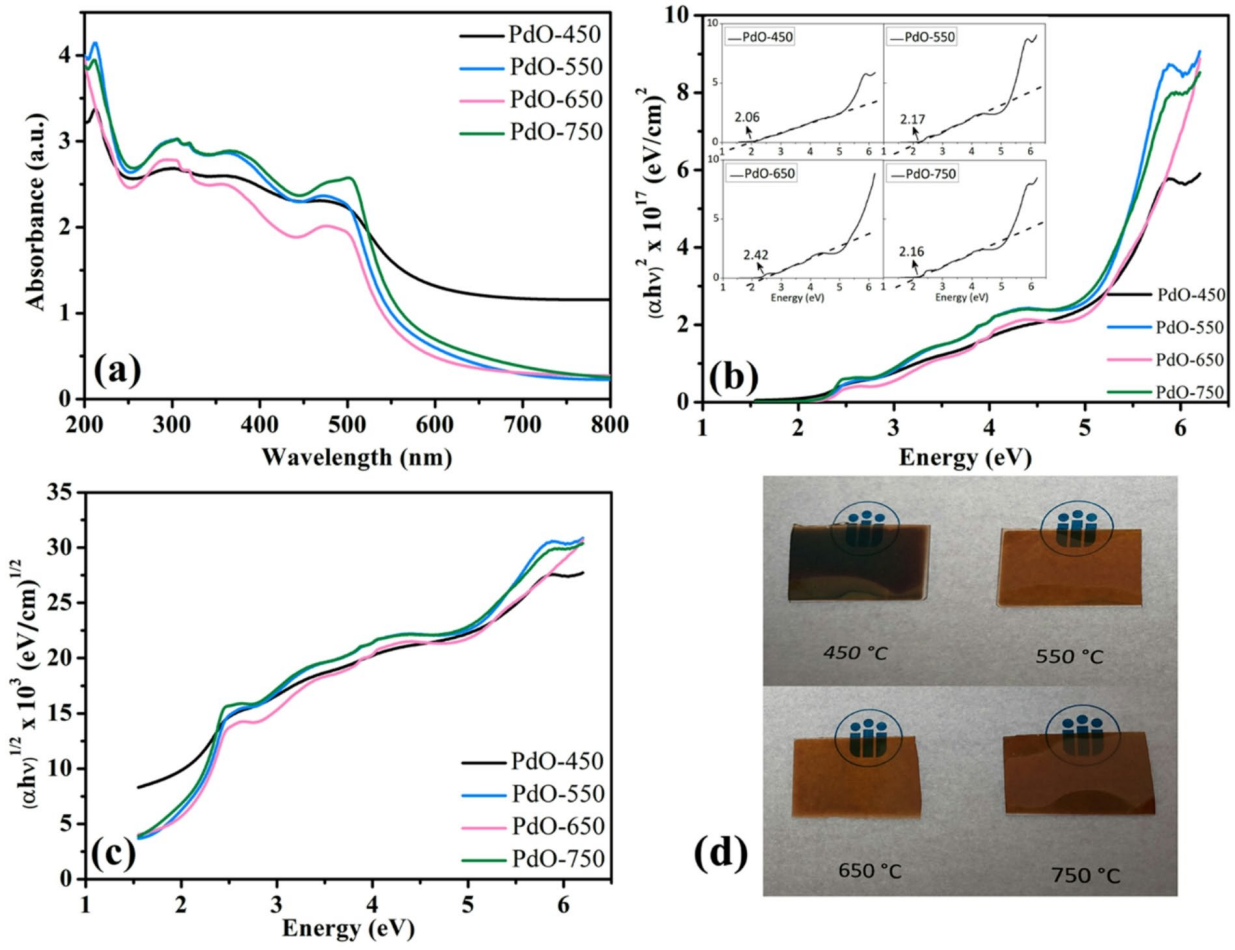
(**a**) UV-Vis absorbance spectra, Tauc plot of (**b**) direct, (**c**) indirect transitions and (**d**) photograph of PdO films. The inset figure in (**b**) show the energy band gap values of the oxidized films.

The optical properties of PdO films were studied using UV-Vis absorption spectra in the wavelength range of 200–800 nm. The significant decrease in absorbance between photon wavelengths of 500–600 nm suggests that there is a decrease in the number of electrons excited from the valence band to the conduction band and acting as free charge carriers. The optical energy band gaps of the PdO films were estimated via a Tauc plot based on the UV-Vis spectra and given by the following relation:

4 $$\:{\left(\alpha\:h\nu\:\right)}^{n}=K(h\nu\:-{E}_{g})$$

where $\:\alpha\:$ is the absorption coefficient, $\:h$ is Planck’s constant, $\:\nu\:$ is the frequency of the radiation, $\:K$ is a proportionality constant, $\:{E}_{g}$ is the band gap energy, and the exponent $\:n$ denotes the characteristic of the electronic transition: for a direct allowed transition, $\:n=2$; for an indirect allowed transition, $\:n=\:1/2$. In Fig. 5b-c, $\:{\left(\alpha\:h\nu\:\right)}^{n}$ versus $\:h\nu\:$ is plotted for both direct and indirect transitions, respectively, to determine the nature of the transition and the magnitude of $\:{E}_{g}$ for each sample. To estimate $\:{E}_{g}$, the most linear portion of the plots was extrapolated to zero, as shown in the inset of Fig. 5b. It can be seen that the linear part of the plots of indirect transitions (Fig. 5c) is not clear enough to make an accurate extrapolation for energies lower than $\:\sim$2.5 eV or for energies higher than $\:\sim$2.5 eV; the intersections with the x-axis occur below zero. Therefore, the main transition in these films is considered to be the direct transition. From Fig. 5b, the $\:{E}_{g}$ of the films were estimated to be 2.06, 2.17, 2.42, and 2.16 eV for PdO-450, PdO-550, PdO-650, and PdO-750 films, respectively, which are close to the results around 2 eV previously obtained by other research groups^34,49^. It is worth underlining that there is a consistency between the magnitudes of the band gap energies and the roughness values given in Fig. 4e, revealing the effect of the crystallization and surface morphology on the band gap energies. Figure 5d shows the photograph of the films just after being exposed to oxygen. It can be seen that the conversion of Pd to PdO changes the color of the film from almost opaque black (which is the color of the Pd films) to transparent brown. PdO-450 reflects the characteristics of both Pd and PdO with a partially transparent structure.

**Fig. 6:**
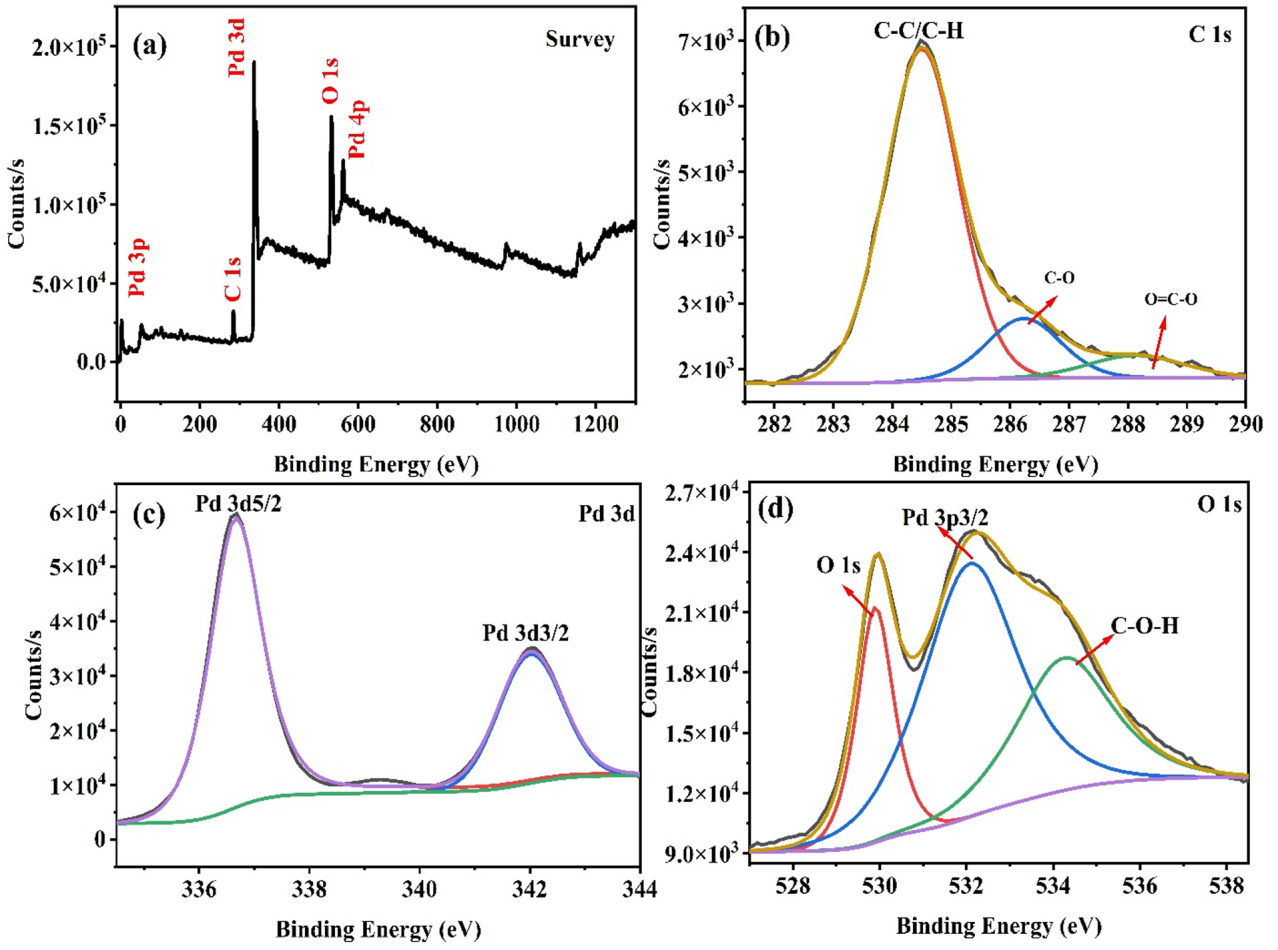
XPS spectra with multicomponent fits for PdO-750 film (**a**) Total survey spectrum, (**b**) the C 1s region, (**c**) Pd 3d region where each spin-orbit split line comprises 2 chemically shifted components, and (**d**) the Pd 3p3/2–O 1s ‘doublet’.

X-ray photoelectron spectroscopy is an important and useful tool for confirming the exact valence states of various elements as well as their chemical composition in the obtained PdO-750 film. The XPS spectrum of PdO-750 is represented in Fig. 6a–d. As revealed in the fully scanned XPS spectra shown in Fig. 6a, the presence of all essential elements (such as Pd 3d, O 1s, and C 1s) was confirmed in the PdO-750 film. The deconvoluted XPS spectra of Pd 3d indicated two significant signals for Pd 3d3/2 and Pd 3d5/2 spin-orbit couplings. In particular, the peaks situated at binding energies of 336.6 and 342 eV are attributed to PdO^50,51^. Notably, the Pd 3d5/2 peak exhibits an upward shift to around 336.6 eV compared to metallic Pd, which typically appears near 335.4 eV. Such a shift of more than 1 eV upon oxidation is consistent with previously reported values in the literature, further confirming the formation of PdO as the dominant chemical state^50,52^. The XPS scan of the C 1s core level comprised three major signals, as depicted in Fig. 6b. Usually, the peaks are situated at high binding energies, including 284.5 and 286.2 eV. As shown in Fig. 6d, the high-resolution X-ray photoelectron spectrum of the O 1s region reveals three distinct components, indicating the chemical states of oxygen in the PdO thin films. The dominant peak centered at 529.8 eV is assigned to lattice oxygen, O²⁻, in the Pd–O bonding environment, confirming the formation of stoichiometric PdO. A secondary peak observed at approximately 532.08 eV is attributed to the Pd 3p3/2 core level, which partially overlaps with the O 1s region. This assignment is consistent with previous studies on Pd-based oxides, where the Pd 3p3/2 signal appears near 531.5 eV and can influence O 1s spectral interpretation^50^. The third peak, a weaker feature located at 534.3 eV, is ascribed to oxygen atoms in carboxyl groups (–COOH) adsorbed on the film surface. The presence of such surface species is likely due to ambient hydrocarbon contamination, as commonly encountered in air-exposed samples. This interpretation is further supported by the appearance of a peak at 289.0 eV in the C 1s spectrum, characteristic of O–C = O bonding environments. The overall spectral profile aligns well with literature reports on PdO thin films and validates the successful oxidation of Pd under the applied annealing conditions^51,53,54^. The above results collectively allow us to discuss the mechanism responsible for PdO formation during thermal oxidation. The oxidation of Pd thin films into PdO may involve both inward oxygen diffusion and outward Pd diffusion, as described by classical diffusion-controlled oxidation models. Based on the combined XRD, FESEM/AFM, and XPS analyses, the present results indicate that oxide growth is predominantly governed by inward oxygen incorporation. The absence of interfacial void formation or delamination, together with the formation of compact and homogeneous PdO layers and the dominance of lattice oxygen in the XPS O 1 s spectrum, suggests that oxygen diffusion through defect-assisted pathways in the growing oxide layer plays the primary role in phase transformation. Although Pd diffusion at elevated temperatures cannot be completely ruled out, the experimental evidence supports inward oxygen diffusion as the dominant contribution to PdO growth under the applied oxidation conditions.

**Fig. 7:**
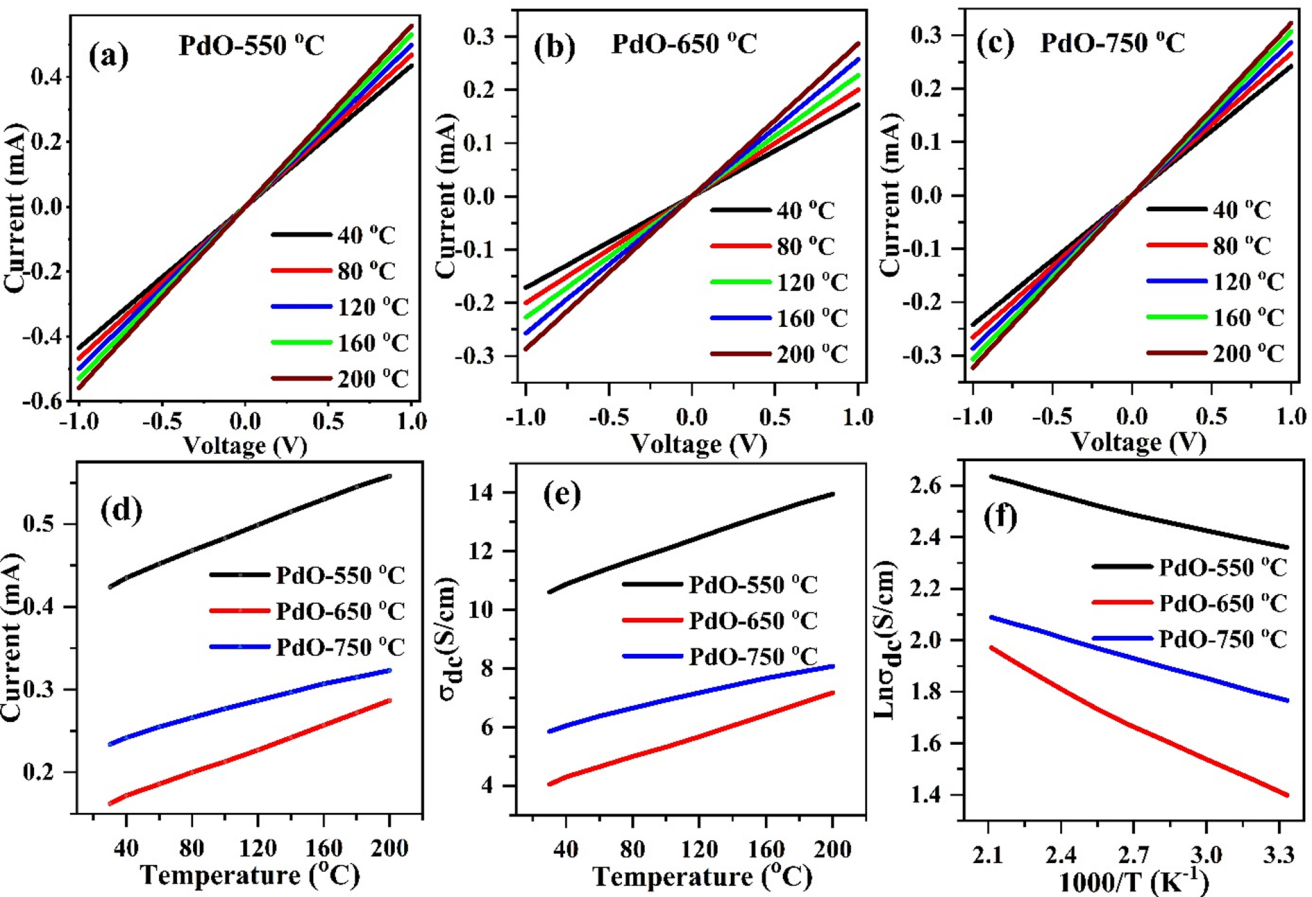
The I-V characteristics under dry air conditions for (**a**) PdO-550, (**b**) PdO-650, (**c**) PdO-750, and (**d**) I-T characteristics of a PdO sample at the indicated temperatures for all samples. (**e**) The DC conductivities of the PdO samples versus temperature, and (**f**) the variation of logarithmic conductivity, lnσdc, with inverse temperature, 1000/T, for the PdO samples.

The current-voltage (I–V) characteristics of PdO thin films, annealed at 550 °C, 650 °C, and 750 °C, are shown in Fig. 7a-c. All samples exhibit a monotonic increase in current with rising temperature under a constant applied voltage, indicative of thermally activated conduction mechanisms. The linear trend in the I–V curves suggests ohmic contact behavior, with no observable non-linearity or rectification effects across the studied temperature range. This observation is consistent with previous reports where ohmic conduction has been attributed to appropriate electrode selection and good interfacial contact, such as the gold contacts used by Rao et al. on PdO thin films^55^. The highest current in the measured voltage interval was observed for the sample PdO-550, which may indicate a higher density of thermally activated carriers or a more favorable defect structure promoting charge transport. As temperature increases, enhanced carrier mobility and thermal excitation over shallow potential barriers contribute to increased conductivity. In Fig. 7d, the current–temperature (I-T) characteristics of the PdO samples are given, with a drive voltage of 1 V. To investigate thermally activated conduction, DC conductivities were calculated and plotted as a function of temperature in Fig. 7e and as an Arrhenius plot in Fig. 7f. The extracted activation energies were found to be 44.8, 92.1, and 52.7 meV for PdO-550, PdO-650, and PdO-750 samples, respectively. These relatively low values indicate a weak thermal activation process, particularly for the sample annealed at 550 °C. The higher activation energy at 650 °C suggests more localized carriers or increased trap density, while the slightly lower activation energy at 750 °C may reflect enhanced crystallinity or improved carrier mobility due to structural rearrangements during annealing. The temperature dependence of the conductivity is well-described by the following equation:

5 $$\:{\sigma\:}_{dc}={\sigma\:}_{0}{e}^{\frac{-{E}_{a}}{{k}_{B}T}}$$

Here, $\:{E}_{a}\:$is the activation energy, $\:{k}_{B}\:$is Boltzmann’s constant, and T is the absolute temperature. The difference in current magnitude among samples annealed at different temperatures suggests variations in defect density, crystallinity, or grain boundary structure, all of which can influence carrier transport. Subsequent analysis of the DC conductivity and Arrhenius plots confirms thermally activated behavior, and activation energies can be extracted to better understand the conduction pathways. Such temperature-dependent trends are common in polycrystalline oxide films, where ionic vacancies or interfacial effects modulate the conduction process^55,56^.

**Fig. 8:**
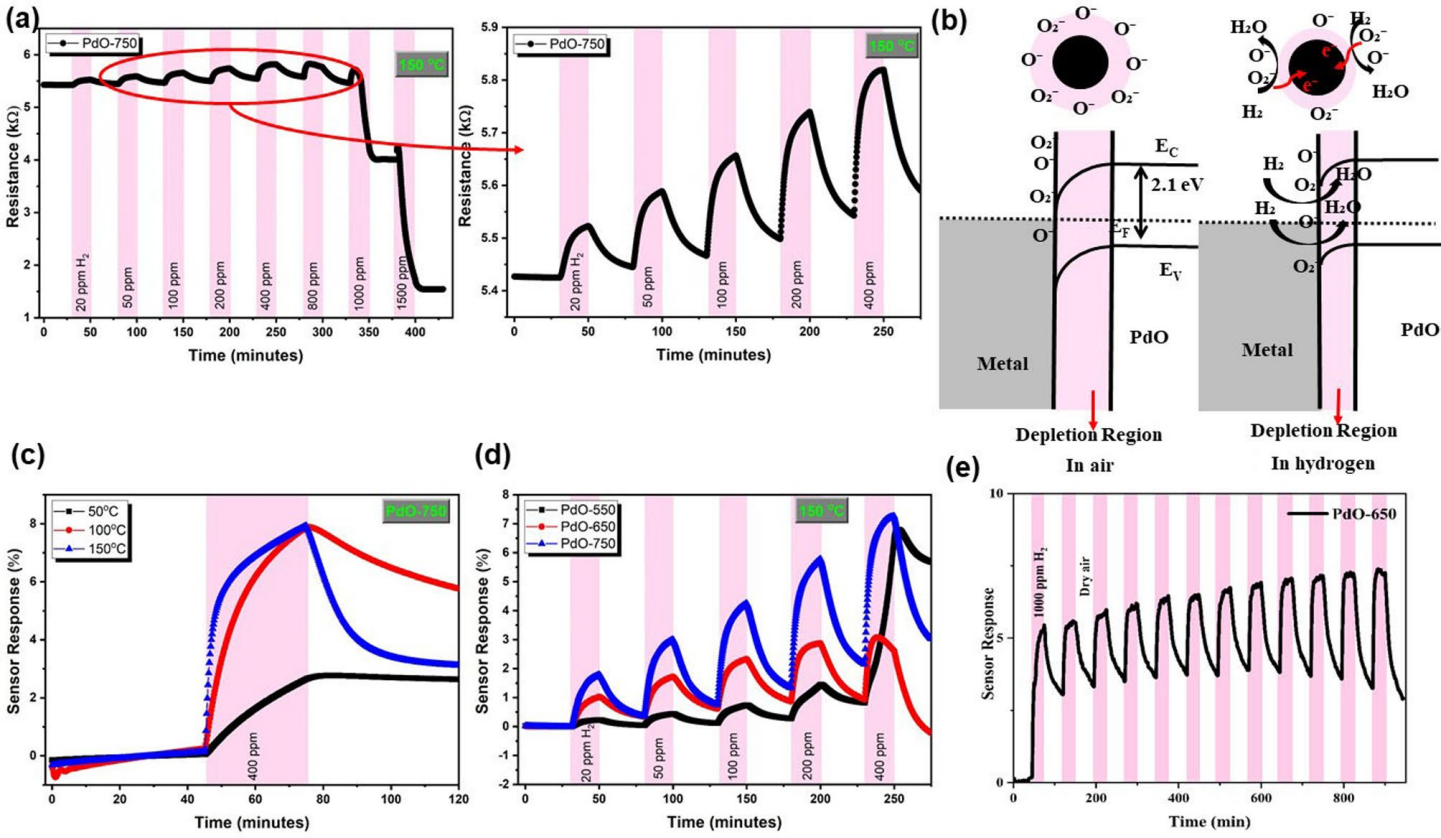
(**a**) Resistance versus time of the 60 nm thick PdO-750 film sample exposed to various hydrogen concentrations from 20 ppm to 1500 ppm at 150 °C, and (**b**) a schematic illustration of the energy band diagram and chemical reactions for hydrogen sensing mechanism. (**c**) The sensor response versus time of the 60 nm thick PdO-750 film sample exposed to 400 ppm hydrogen at various temperatures. (**d**) The sensor responses of the 60 nm thick PdO films as a function of time exposed to the indicated hydrogen concentrations at 150 ºC. (**e**) Sensor responses of the  60 nm thick PdO-650 sample exposed to 1000 ppm H₂ and dry air in cyclic intervals at room temperature.

As evidenced by the sensing characteristics in our measurements, the hydrogen detection mechanism of PdO operates through two distinct pathways: one is a conventional p-type semiconductor response that dominates at low hydrogen concentrations, where surface oxygen reactions modulate the hole-accumulation layer, and the other is a reduction-driven mechanism that becomes active under high hydrogen concentrations, leading to the chemical transformation of PdO into metallic Pd. These two regimes together define the dual sensing behavior observed in PdO-based hydrogen sensors. To investigate the conventional p-type semiconductor hydrogen sensing properties of the PdO, it was first stabilized under a flow of dry air at various temperatures to establish a baseline resistance and then exposed to indicate hydrogen concentrations while the change in resistance was recorded. Measurements were performed for both low hydrogen concentrations and a high concentration (2%). The hydrogen concentrations were set by diluting hydrogen with dry air using mass flow controllers, and the gas mixture was allowed to flow over the sensor during each H₂ exposure; recovery was monitored by switching back to dry air for a constant time. The sensor displayed reproducible response–recovery cycles, with the sensor response defined as follows:

6 $$\:Response=\:\frac{{R}_{H}-{R}_{air}}{{R}_{air\:}}x100$$

The hydrogen sensing behavior of the 60 nm thick PdO-750 film sample at 150 °C (Fig. 8a) is explained by the surface oxygen-driven modulation of the hole accumulation layer, consistent with previous reports on p-type PdO^57,58^. In dry air, as shown in Fig. 8b, chemisorbed oxygen species extract electrons from the PdO surface, generating an upward band bending and forming a hole-accumulation layer that provides a low-resistance conduction path. When the sensor is periodically switched from dry air to low-concentration hydrogen, the reducing gas reacts with the adsorbed oxygen and partially depletes these surface species, allowing some trapped electrons to recombine with holes. As a consequence, the hole accumulation layer becomes thinner, leading to an increase in resistance^58^. The higher work function of PdO (∼5.8–5.9 eV) compared to most contacting metals leads to an upward band bending at the interface, creating a surface depletion/accumulation region relative to the surrounding environment, which should affect the hydrogen sensing mechanism. In dry air, oxygen-induced electron withdrawal increases this band bending and stabilizes the hole accumulation layer. Under hydrogen exposure, depletion of surface oxygen weakens this band bending, narrows the accumulation region, and increases the overall resistance of the PdO layer. The resistance of the PdO-750 sample increases in the hydrogen environment up to a hydrogen concentration of 800 ppm and then decreases during cleaning with dry air, as seen in Fig. 8a. When 1000 ppm and 1500 ppm hydrogen were exposed to the PdO-750 sample, the resistance of the sample increased rapidly and then decreased sharply. This indicates that the reduction process begins by exposure to 1000 ppm hydrogen at a temperature of 150 °C, and the beginning of the reduction process needs higher hydrogen concentrations at room temperature. Yang et al. prepared Pd-decorated PdO nanoparticle nanonetwork films and measured tunable color change in the hydrogen concentration range of 0.5% to 100%^59^. Figure 8d shows the sensor response-time graph of 60 nm thick PdO films annealed at different temperatures (550 °C, 650 °C and 750 °C) against different hydrogen concentrations at the operation temperature of 150 °C. It is observed that the sensor response increases with increasing annealing temperature. XRD results indicate that the crystallinity of the PdO film increases with annealing temperature, and this situation correlates with the hydrogen response. This increased crystallinity may affect the mobility of charge carriers in the PdO semiconductor film, potentially leading to a better charge transfer process during hydrogen detection. To examine the effect of temperature on sensor performance, test results obtained at 50 °C, 100 °C, and 150 °C when the PdO-650 thin film sample was exposed to 400 ppm hydrogen are given in Fig. 8c. It is observed that the sensor response increases with increasing temperature, and the sensor responses at 100 °C and 150 °C are almost the same value. Furthermore, it is observed that the sensor response is very slow at low temperatures and does not return to the baseline value. In conclusion, it is seen that the PdO-650 thin film sensor can operate at 100 °C and higher temperatures. To enable PdO thin films to operate at low temperature, even at room temperature, Pt was used as the contact electrode, and as shown in Fig. 8e, a repeatable response was obtained when 12 times cycles of 1000 ppm hydrogen-dry air were exposed to the PdO-650 thin film sample at room temperature. Replacing the Ag electrode with Pt enhances the catalytic activity at the sensor interface, promoting efficient electron transfer and accelerating the hydrogen oxidation reaction on the surface. Consequently, the resulting device architecture exhibits strong potential for the development of high-performance, room-temperature hydrogen sensors^60,61^. The periodic recovery in air further confirms that the sensing process is fully reversible and governed solely by oxygen adsorption-desorption dynamics, not by structural reduction to metallic Pd^62^.

**Fig. 9:**
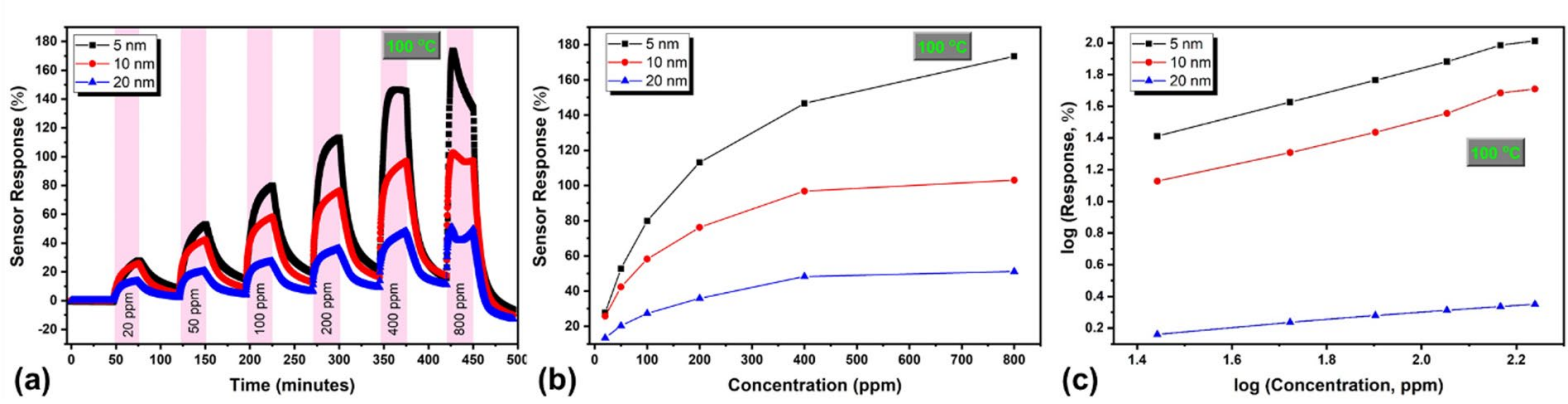
Effect of thickness on performance of PdO thin film hydrogen sensor: (**a**) Sensor response versus time, (**b**) concentration dependent sensor response, and (**c**) log (sensor response) versus log (concentration) graphs for 650 °C annealed PdO thin films with different thicknesses exposed to various hydrogen concentrations from 20 ppm to 800 ppm at 100 °C.

In order to evaluate the effect of thickness on hydrogen sensing performance, 650 °C annealed PdO thin film with various thicknesses (5, 10, and 20 nm) were tested under low hydrogen concentrations. Figure 9a presents the sensor response of PdO thin films exposed to low hydrogen concentration (20–800 ppm) at 100 °C and indicates the increase in the sensor response with decreasing film thickness as expected. Furthermore, it is observed that the sensor response increases with increasing hydrogen concentration, and Fig. 9b shows the concentration-dependent change in the sensor response of PdO films with different thicknesses. The figure shows a nonlinear increase between sensor response and concentration. The hydrogen adsorption kinetics of PdO films can be explained by Freundlich’s adsorption isotherm, and Fig. 9c gives the linear behavior between logarithmic sensor response and hydrogen concentration as evidence for this isotherm. This isotherm was proposed for multilayer adsorption and applied to heterogeneous surface sites over a small concentration range^63,64^. The proposed mathematical model for this model can be written as follows:

7 $$\:{Sensor\:Response\:\approx\:q}_{e}={K}_{F}{{C}_{e}}^{1/n}$$

Where q_e_ is the adsorbed analyte amount at equilibrium, 1/n is the coefficient of surface heterogeneity, and K_F_ is the absorption capacity coefficient. The state of Freundlich’s absorption isotherm process could be explained based on the 1/n value, and for 1/n values between 0 and 1 and equal to 1 and greater than 1, the adsorption process can be described as favorable, irreversible, and unfavorable, respectively. In our case, 1/n values of 5 nm, 10 nm, and 20 nm PdO thin films are observed as 0.77, 0.75, and 0.24 respectively. Therefore, Freundlich’s absorption isotherm process could be suitable for PdO thin-film resistive hydrogen sensors.

**Fig. 10:**
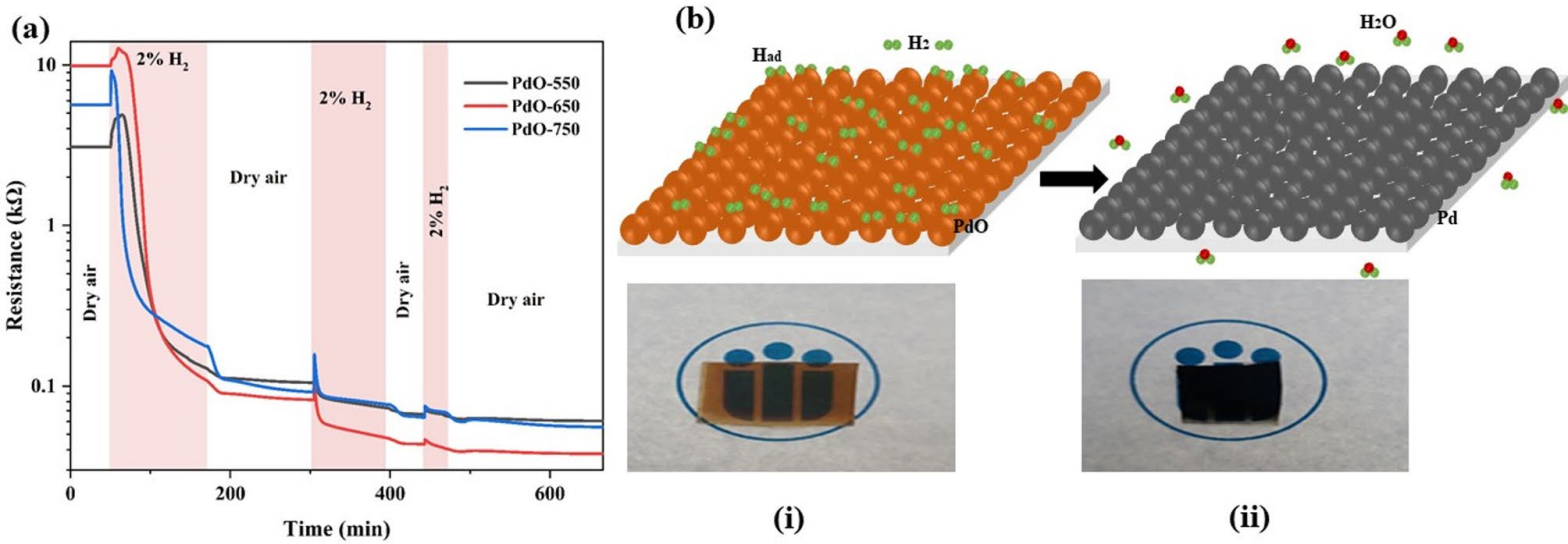
(**a**) The resistance characteristics of PdO thin films annealed at 550 °C, 650 °C, and 750 °C, measured under 2% H_2_. (**b**) Schematic illustration and photographs of H_2_ adsorption and desorption. (i) Exposure of hydrogen molecules and adsorption of hydrogen molecules and dissociation of hydrogen atoms (H_ad_) on PdO (ii) The conversion of PdO to Pd on PdO and desorption of water molecules.

To evaluate the other reduction-driven mechanism that activates PdO films under high H₂ concentrations in hydrogen environments above 1000 ppm, sensor response measurements were carried out at room temperature in alternating dry air, as shown in Fig. 10a. The claim that there is a color difference resulting from the reduction of PdO to Pd as a result of contact with hydrogen gas at a concentration higher than 1000 ppm is also supported by various literature providing information about the mechanism of colorimetric hydrogen sensors^65–68^. The mechanism of colorimetric hydrogen sensing by PdO can be described as follows:

8 $$\:PdO+{H}_{ad}\to\:PdO-H$$

9 $$\:PdO-H+{H}_{ad}\to\:Pd+{H}_{2}O$$

Here, $\:{H}_{ad}$ is an adsorbed hydrogen atom. When the PdO sensor is exposed to hydrogen gas, hydrogen molecules adsorb onto the PdO and subsequently dissociate into hydrogen atoms on the $\:PdO$ surface, forming $\:PdO-H$. The adsorbed hydrogen atoms then transform $\:PdO-H$ into $\:Pd$ through the overflow effect^66,67^. The first stage is the induction period for color deterioration and is affected by the hydrogen concentration. The second stage, the color deterioration stage, is affected by the PdO-H concentration, and this color change is shown in Fig. 10b (i) and (ii). At such high H₂ concentrations, the surface reactions go beyond simple oxygen consumption and instead trigger a chemical reduction of PdO to metallic Pd. This transformation produces a dramatic change in the electronic structure and, consequently, in the resistance of the sensor.

**Fig. 11:**
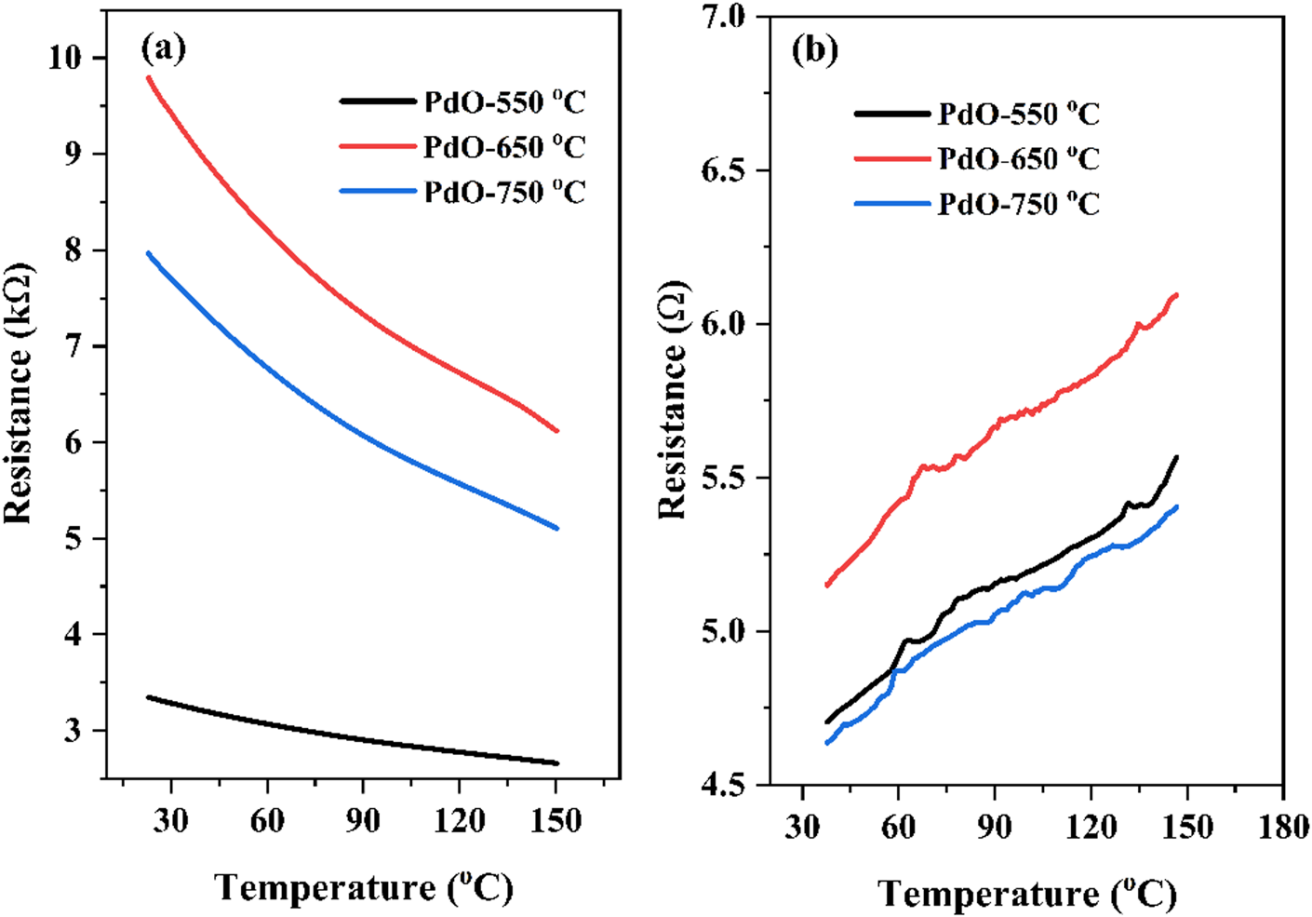
Temperature-dependent resistance characteristics of PdO thin films before and after exposure to high-concentration H₂ (2%). (**a**) Resistance of pristine PdO films annealed at 550 °C, 650 °C, and 750 °C measured in dry air before H₂ exposure. (**b**) Temperature-dependent resistance characteristics of the same PdO thin films annealed at 550 °C, 650 °C, and 750 °C after exposure to 2% H_2_.

The temperature-dependent resistance behavior of PdO thin films before and after exposure to high-concentration H₂ (2%) is shown in Fig. 11a, b. All measurements were performed in dry air environment with a multimeter. In Fig. 11a, all PdO samples exhibit a decrease in resistance with increasing temperature, which is a typical behavior for p-type semiconducting metal oxides. Among the samples, the film annealed at 550 °C exhibited the lowest resistance variation, while the 650 °C sample showed the highest resistance across the measured temperature range. The observed temperature-resistance relationship is consistent with the behavior of polycrystalline PdO, where annealing affects the crystallinity, grain boundary density, and intrinsic defect concentrations. Similar findings were reported by Rogers et al., who observed semiconducting conductivity in PdO crystals with a low thermal activation energy attributed to extrinsic charge transport mechanisms^69^. Furthermore, Shibasaki and Terasaki demonstrated that layered Pd-based oxides exhibit activation energies ranging from 70 to 80 meV, depending on carrier localization and lattice disorder^56^. However, after exposure to high-concentration hydrogen (2%), the electrical behavior transitions to a metallic-like regime in which the resistance becomes markedly lower and displays a weak positive temperature coefficient. This change indicates the reduction of PdO to metallic Pd, where electron–phonon scattering dominates the transport mechanism. After the chemical reduction to Pd under high H₂ concentrations, which dramatically enhances conductivity. This dual behavior aligns with previously reported PdO-based hydrogen sensing mechanisms and supports the reduction-driven response observed in this study^47,70,71^.

## Conclusion

A systematic study was carried out to understand the effects of elemental chemical states and structural phases in PdO thin films on their electrical and optical properties. PdO thin films were fabricated by sputter deposition of Pd followed by thermal oxidation at different annealing temperatures. Structural analyses confirmed that complete Pd-to-PdO conversion occurs above 550 °C, with crystallinity and domain size improving at higher temperatures. XRD and XPS survey scans and deconvolution of high-resolution scans were used to identify the presence of PdO. AFM and FESEM revealed that oxidation strongly influences the surface morphology, producing the highest porosity and roughness at 650 °C, while further annealing at 750 °C led to more compact and crystalline structures. Optical measurements indicated direct band gaps in the range of 2.06–2.42 eV, consistent with the observed microstructural evolution. XPS verified the formation of stoichiometric PdO with Pd²⁺ oxidation states and minor surface-related oxygen species. Electrical characterizations showed thermally activated semiconducting behavior, with activation energies between 45 and 92 meV, reflecting the role of defects and grain boundaries in carrier transport. Hydrogen sensing measurements were carried out at room temperature and under varying operating temperatures to assess thermal stability. The sensor response showed no significant dependence on operating temperature, indicating that the sensing mechanism remains largely temperature-independent within the investigated range. Among the investigated samples, the film annealed at 750 °C exhibited the highest sensing response at 400 ppm H₂, outperforming those annealed at lower temperatures. Furthermore, replacing the Ag electrode with Pt markedly enhanced the sensing performance owing to the superior catalytic activity of Pt. The Pt-contacted device reliably detected 1000 ppm H₂ at room temperature, demonstrating stable operation without the need for elevated operating temperatures. Importantly, the room temperature hydrogen sensing experiments revealed that PdO operates through two distinct sensing regimes. At low H₂ concentrations (≤ 1000 ppm), the response is governed by a conventional p-type surface-controlled mechanism, in which H₂ reacts with chemisorbed oxygen and modulates the hole-accumulation layer, leading to reversible resistance changes. In contrast, at high hydrogen concentrations (2%), PdO undergoes a chemical reduction to metallic Pd, resulting in a sharp drop in resistance and a transition to metallic-like temperature dependence. This transformation was also supported by colorimetric changes.

## Data Availability

The datasets generated and analyzed during the current study are available from the corresponding author on reasonable request.
